# Current insights into renal ciliopathies: what can genetics teach us?

**DOI:** 10.1007/s00467-012-2259-9

**Published:** 2012-07-25

**Authors:** Heleen H. Arts, Nine V. A. M. Knoers

**Affiliations:** 1 Department of Human Genetics, Nijmegen Centre for Molecular Life Sciences, and Institute for Genetic and Metabolic Disease, Radboud University Nijmegen Medical Centre, 6525 GA Nijmegen, The Netherlands; 2 Department of Medical Genetics, University Medical Center Utrecht, 3508 AB Utrecht, The Netherlands

**Keywords:** Cilia, Renal ciliopathies, Renal cysts, Genotype–phenotype correlations, Next-generation sequencing, Personalized medicine

## Abstract

Ciliopathies are a group of clinically and genetically overlapping disorders whose etiologies lie in defective cilia. These are antenna-like organelles on the apical surface of numerous cell types in a variety of tissues and organs, the kidney included. Cilia play essential roles during development and tissue homeostasis, and their dysfunction in the kidney has been associated with renal cyst formation and renal failure. Recently, the term “renal ciliopathies” was coined for those human genetic disorders that are characterized by nephronophthisis, cystic kidneys or renal cystic dysplasia. This review focuses on renal ciliopathies from a human genetics perspective. We survey the newest insights with respect to gene identification and genotype–phenotype correlations, and we reflect on candidate ciliopathies. The opportunities and challenges of next-generation sequencing (NGS) for genetic renal research and clinical DNA diagnostics are also reviewed, and we discuss the contribution of NGS to the development of personalized therapy for patients with renal ciliopathies.

## Introduction

### Cilia

**Fig. 1:**
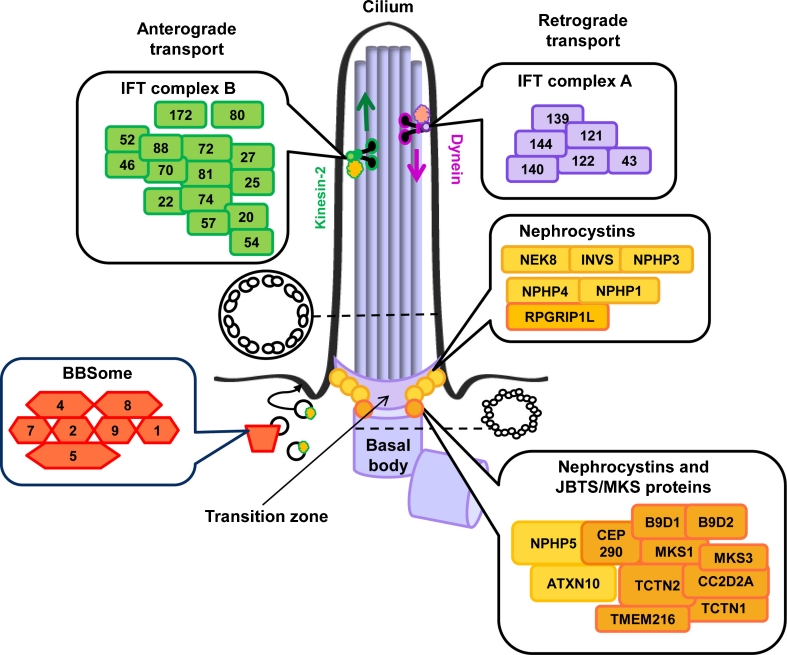
The primary cilium and ciliary protein complexes. The primary cilium is a membrane-enclosed antenna-like structure with a ring-shaped skeleton that consists of 9 doublets of microtubules. The ciliary base is called the “basal body”, and consists of triplets of microtubules. Ciliary transport, intraflagellar transport (IFT), occurs from base-to-tip mediated by the IFT complex B (*green*) in association with a kinesin II motor and from tip-to-base by the IFT complex A (*purple*) in association with the cytoplasmic dynein motor 2. Other protein complexes are the BBSome (*red*) consisting of various BBS proteins, and networks of nephrocystins (*yellow*), and Meckel–Gruber (MKS) and/or Joubert (JBTS) syndrome-associated proteins (*orange*). The BBSome is involved in trafficking membrane proteins to the cilium, while most nephrocystins and MKS/JBTS proteins localize to the transition zone where they are important for ciliogenesis, regulation of ciliary signaling and the docking and filtering of vesicles/proteins at the cilium

Cilia are membrane-enclosed hair-like cell organelles that occur on the apical surface of renal tubular cells and on cells in many other organs. Cilia are conserved among species and were first described by the Dutch scientist Antoni van Leeuwenhoek, the father of microscopy and cell biology, who reported ciliated micro-organisms that used their cilia as “little legs” for movement [1, 2]. It appears that many cell types in the human body, such as sperm cells and the respiratory epithelial cells, also contain motile cilia. The cilium, or flagellum, of a sperm cell allows the cell to move, whereas cilia in the respiratory system propel mucous over the cell surface [3]. In this review, we will focus on the immotile brother of the motile cilium, the so-called primary cilium, which appears and functions as a cell antenna of renal cells and cells throughout the human body (Fig. 1) [3, 4]. Structurally, the primary cilium is composed of a basal body from which the cilium initially assembles, a transition zone that is important for anchoring the cilium to the membrane and regulating protein traffic in and out of the cilium, and the ciliary axoneme, which contains a ring of microtubule bundles connecting the ciliary base with the tip. The microtubules form the skeleton of the cilium and are literally a “highway” for ciliary transport (intraflagellar transport, or IFT), a process that was first observed in the unicellular green alga *Chlamydomonas reinhardtii* and that has recently been extensively reviewed by Ishikawa and Marshall [5, 6]. This transport process is bidirectional, base-to-tip (anterograde) and tip-to-base (retrograde), and occurs through interactions of the kinesin-2 motor in association with the IFT complex B proteins or the cytoplasmic dynein motor 2 linked to IFT complex A proteins respectively [5]. IFT allows movement of cargo through the cilium and is important for ciliogenesis and for signaling cascades that regulate development and tissue homeostasis [3, 4].

### Ciliary dysfunction and renal insufficiency

One of the first papers that linked ciliary disruption to the development of cystic kidneys in mammals was published by the group led by Douglas Cole in 2000. In their paper they describe ciliary abnormalities and renal disease in a mouse model with a hypomorphic mutation in the *Ift88* gene that encodes a protein that is part of the IFT B complex (Fig. 1) [7]. To date, we know that ciliary disruption is linked to a variety of human genetic kidney disorders, such as autosomal dominant and recessive polycystic kidney disease (ADPKD and ARPKD), tuberous sclerosis (TSC), medullary cystic kidney disease (MCKD), and nephronophthisis and related disorders [3, 4]. Here, we will predominantly focus on the latter group of disorders.

## Renal ciliopathies

### Nephronophthisis

**Fig. 2:**
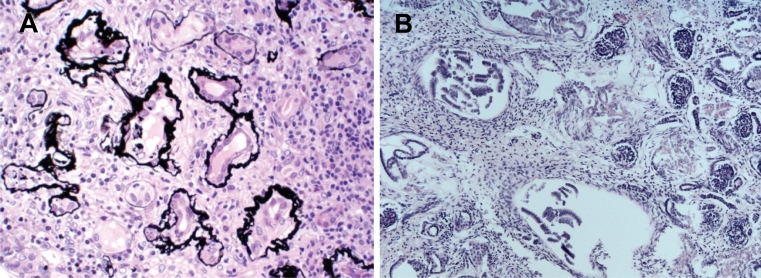
Nephronophthisis and renal cystic dysplasia. **a** Pathohistology of nephronophthisis. A cross section through a renal biopsy from a Sensenbrenner patient shows interstitial fibrosis and tubular membrane disruptions (thickened, irregular basement membranes). Image courtesy of Eric Steenbergen. **b** Cystic dysplastic kidneys with marked interstitial fibrosis and cysts of different sizes form in a fetus with Meckel–Gruber syndrome. Image courtesy of Carsten Bergmann

Nephronophthisis literally means “damage to the nephrons.” It is an autosomal recessive disorder that represents the most common monogenetic cause of renal insufficiency in children and young adults. It is enormously genetically heterogeneous, i.e., mutations in at least 13 different genes have been associated with nephronophthisis. In spite of that, 70% of patients still remain genetically unexplained [8]. In 1997, the first genetic cause of nephronophthisis was identified through the detection of a deletion that covered the *NPHP1* gene [8, 9]. Later, it became apparent that *NPHP1* is not only mutated in isolated nephronophthisis, but that a significant number of patients with *NPHP1* mutations also display neurological symptoms. Some of these individuals have been reported to display cerebellar vermis hypoplasia and brainstem anomalies compatible with a Joubert syndrome diagnosis [10–12]. With respect to nephronophthisis, *NPHP1* is the most commonly mutated gene, as genetic defects in this gene explain the cause of disease in 20% of patients with this disorder [8], while all other nephronophthisis-associated genes (Table 1) have been found to be mutated with a much lower frequency. Remarkably, almost all of these genes encode proteins that interconnect in a dynamic “nephrocystin” protein complex that resides at the transition zone (Fig. 1) where it regulates ciliogenesis and protein sorting, thereby controlling renal development and homeostasis [13–15]. Yet, other localizations and functions of the nephrocystins are also known. Besides their ciliary roles, nephrocystins 1, 4, and RPGRIP1L (also known as nephrocystin-8) have been shown to regulate tight-junction formation at the cell junctions [16]. GLIS2 and nephrocystin-2 function in both the nucleus and the cilium [17, 18], and the recently identified XPNPEP3 biochemically processes several ciliary proteins and has been detected in mitochondria [19]. Clinically, it is difficult to diagnose nephronophthisis in early stages of the disease as children with this rare disorder initially present with nonspecific features such as polydipsia and polyuria [8]. As such, good medical care for patients with nephronophthisis (and other cystic kidney diseases) includes evaluation for other medical and developmental issues. Ultrasound, renal biopsies, and/or genetic tests are necessary to make a definite nephronophthisis diagnosis. Renal ultrasounds often show normal sized or small kidneys with increased echogenicity, and may reveal renal cysts, although cysts are not recorded in all nephronophthisis patients. Histologically, nephronophthisis is characterized by thickened and irregular tubular basement membranes, periglomerular and interstitial fibrosis, and (sporadic) cysts that often occur at the corticomedullary border (Fig. 2a) [8].

**Table 1:** Ciliary disease genes and renal phenotypes

Symbol	Renal phenotype in patients	MIM Gene ID	Disorders	Reference (PMID)
*AHI1*	Nephronophthisis	608894	JBTS	15322546
*ALMS1*	Renal insufficiency	606844	ALSTR	11941369; 11941370
*ARL13B*	No renal disease reported	608922	JBTS	18674751
*ARL6*	Renal failure, kidney stones	608845	BBS, RP	15258860; 15314642; 19858128; 19956407
*ATXN10*	Nephronophthisis	611150	NPHP	21565611
*B9D1*	Multicystic dysplastic kidneys	614144	MKS	21493627
*B9D2*	Cystic kidneys	611951	MKS	21763481
*BBS1*	Chronic renal failure, urinary tract infections and anomalies	209901	BBS	12118255
*BBS10*	Meckel-like cystic kidneys	610148	BBS	16582908
*BBS12*	Renal disease reported	610683	BBS	17160889
*BBS2*	Meckel-like cystic kidneys, cystic kidneys, renal hypoplasia	606151	BBS	11285252
*BBS4*	Meckel-like cystic kidneys, cystic kidneys	600374	BBS, LCA	11381270
*BBS5*	No renal disease reported	603650	BBS	15137946
*BBS7*	Renal disease reported	607590	BBS	12567324
*BBS9*	Renal disease reported	607968	BBS	16380913
*CC2D2A*	Cystic dysplastic kidneys, nephronophthisis	612013	COACH, JBTS, MKS	19574260; 18387594; 18513680; 18950740
*CEP41*	Nephronophthisis (rare)	610523	JBTS	22246503
*CEP290*	Multicystic dysplastic kidneys, nephronophthisis	610142	BBS, JBTS, MKS, SLSN, LCA	17617513; 17564974; 18327255; 16682970; 16682973; 16909394; 21068128
*C5ORF42*	No renal disease reported	614571	JBTS	22425360
*DYNC2H1*	Cystic kidneys, Multicystic dysplastic kidneys	603297	ATD, SRP	19442771
*EVC*	No renal disease reported	604831	EVC	10700184
*EVC2*	No renal disease reported	607261	EVC	12468274
*GLIS2*	Nephronophthisis	608539	NPHP	17618285
*HYLS1*	No renal disease reported	610693	HYLS	15843405; 18648327
*IFT122*	Nephronophthisis	606045	CED	20493458
*IFT43*	Nephronophthisis	614068	CED	21378380
*IFT80*	No renal disease reported	611177	ATD, SRP	17468754; 19648123
*IFT140*	Nephronophthisis	614620	ATD, SM	22503633
*INPP5E*	No renal disease reported	613037	JBTS, MORM	19668215; 19668216
*INVS*	Enlarged (dysplastic) cystic kidneys	243305	NPHP, SLSN	12872123; 16522655
*IQCB1*	Nephronophthisis	609237	SLSN, LCA	15723066; 21220633
*KIF7*	No renal disease reported	611254	ACRC, HYLS, JBTS	21633164; 21552264
*MKKS*	Meckel-like cystic kidneys, lobulated kidneys	604896	BBS, MKKS	10973251; 10973238
*MKS1*	Renal cystic dysplasia	609883	MKS	16415886
*NEK1*	Cystic kidneys, horseshoe kidney (rare)	604588	SRP	21211617
*NEK8*	Nephronophthisis	609799	NPHP	18199800
*NPHP1*	Nephronophthisis	607100	NPHP, JBTS, SLSN	9326933; 15138899; 9856524
*NPHP3*	Nephronophthisis, renal cystic dysplasia	608002	NPHP, MKS, SLSN	12872122; 18371931; 11752023
*NPHP4*	Nephronophthisis	607215	NPHP, SLSN	12205563; 12244321
*OFD1*	Cystic kidneys	300170	JBTS, OFD, SGBS	11179005; 19800048; 16783569
*OCRL1*	Renal proximal tubulopathy (Dent’s disease)	300535	OCRL	22228094
*PKD1*	Enlarged cystic kidneys	601313	ADPKD	8004675
*PKD2*	Enlarged cystic kidneys	173910	ADPKD	8650545
*PKHD1*	Enlarged cystic kidneys	606702	ARPKD	11898128; 11919560
*RPGRIP1L*	Multicystic dysplastic kidneys, enlarged cystic kidneys, nephronophthisis	610937	COACH, JBTS, MKS	17558407; 17558409; 19574260
*SDCCAG8*	Nephronophthisis	613524	SLSN, BBS	20835237; 22190896
*TCTN1*	No renal disease reported	609863	JBTS	21725307
*TCTN2*	Enlarged cystic kidneys	613846	JBTS, MKS	21565611; 21462283
*TMEM138*	Renal cystic dysplasia, nephronophthisis	614459	JBTS	22282472
*TMEM237*	Cystic kidneys	614423	JBTS	22152675
*TMEM216*	Renal cystic dysplasia, cystic kidneys, nephronophthisis	613277	JBTS, MKS	20036350; 20512146
*TMEM67*	Renal cystic dysplasia, (micro)cystic kidneys, nephronophthisis	609884	COACH, JBTS, MKS, NPHP	19058225; 17160906; 16415887; 19508969
*TRIM32*	No renal disease reported	602290	BBS	16606853
*TTC21B*	Nephronophthisis	612014	ATD, NPHP	21258341
*TSC1*	Cystic kidneys, renal cancer	605284	TSC	9242607
*TSC2*	Cystic kidneys, renal cancer	191092	TSC	7581393
*TTC8*	Renal dysplasia (rare)	608132	BBS, RP	14520415; 20451172
*UMOD*	Renal (glomerulo)cystic disease, interstitial nephropathy	191845	MCKD, FJHN, GCKD	14570709; 12629136; 12471200
*VHL*	Renal cell carcinoma, cystic kidneys	608537	VHL	2894613; 15611513
*WDPCP*	No renal disease reported	613580	BBS	20671153
*WDR19*	Nephronophthisis	608151	ATD, CED, NPHP	22019273
*WDR35*	Cystic kidneys	613602	CED, SRP	21473986; 20817137
*XPNPEP3*	Nephronophthisis	613553	NPHP	20179356

*ADPKD* autosomal dominant polycystic kidney disease, *ALSTR* Alström syndrome, *ARPKD* autosomal recessive polycystic kidney disease, *ATD* asphyxiating thoracic dystrophy, *BBS* Bardet–Biedl syndrome, *CED* cranioectodermal dysplasia, *COACH* cerebellar vermis hypo/aplasia, oligophrenia, ataxia, coloboma and hepatic fibrosis, *EVC* Ellis–van Creveld syndrome, *FJHN* familial juvenile hyperuricemic nephropathy, *GCKD* glomerulocystic kidney disease with hyperuricemia and isosthenuria, *HYLS* hydrolethalus syndrome, *JBTS* Joubert syndrome, *LCA* Leber congenital amaurosis, *MCKD* medullary cystic kidney disease, *MKS* Meckel–Gruber syndrome, *NPHP* nephronophthisis, *OCRL* Lowe oculo-cerebro-renal syndrome, *OFD* oro-facio-digital syndrome, *RP* retinitis pigmentosa, *SGBS* Simpson–Golabi–Behmel syndrome, *SLSN* Senior–Løken syndrome, *SM* Saldino–Mainzer syndrome, *SRP* short rib polydactyly, *Usher* Usher syndrome, *TSC* tuberous sclerosis, *VHL* Von Hippel–Lindau disease, *NA* not available

### Syndromes associated with nephronophthisis

Nephronophthisis is often accompanied by anomalies in other organs (Table 2). This is not surprising given the fact that primary cilia occur almost ubiquitously throughout the human body [4]. Extrarenal features that are often observed include retinal degeneration, hepatobiliary disease, cerebellar vermis hypoplasia, laterality defects, intellectual disability and shortening of bones (ribs, phalanges and long bones) [3, 4]. These features are represented in a variety of syndromes, including Senior–Løken syndrome (retinal degeneration causing blindness), Joubert syndrome (cerebellar vermis hypoplasia and brainstem abnormalities; the primary hallmark is the molar tooth sign in the brain), Bardet–Biedl syndrome (intellectual disability, obesity, and various other features), and Jeune asphyxiating thoracic dystrophy (shortening of the bones, the main characteristic is a narrow rib-cage) [3, 4]. In 2009, it was suggested by Baker and Beales and by Konstantinidou et al that Sensenbrenner syndrome, often but not always characterized by nephronophthisis, is also part of the ciliopathy spectrum based on the phenotypic overlap with the classic ciliopathies [20, 21]. A year later the first genetic evidence was published by us and others supporting this assumption [22, 23]. We can therefore conclude that classification of classic and new ciliopathies allows ciliary gene prioritization facilitating gene hunting for these disorders. Sensenbrenner syndrome is characterized by several of the above-mentioned features, for instance, retinal degeneration, hepatobiliary disease, cerebellar vermis hypoplasia, and shortening of the bones in combination with craniosynostosis and ectodermal anomalies, such as skin laxity and tooth abnormalities [24–26]. To date, Sensenbrenner syndrome and the other ciliary disorders affecting skeleton development, such as Jeune syndrome, Ellis–van Creveld syndrome, Saldino–Mainzer syndrome, and the short-rib-polydactyly syndrome are referred to as “skeletal ciliopathies.”

**Table 2:** Phenotype overlap in renal ciliopathies

	BBS	MKS	JBTS	NP4P	SLSN	OFD1	CED	ATD	SRP	ALSM	PKD
Cystic kidneys	∎	∎	∎	∎	∎	∎	∎	∎	∎	∎	∎
Hepatobiliary disease	∎	∎	∎	∎		∎	∎	∎	∎	∎	∎
Retinal degeneration	∎	∎	∎		∎	∎	∎	∎		∎	
Laterality defects	∎	∎		∎	∎			∎	∎		
Intellectual disability	∎	∎	∎			∎		∎			
Cerebellar vermis hypoplasia			∎			∎	∎	∎	∎		
Encephalocele		∎	∎								
Polydactyly	∎	∎	∎			∎	∎	∎	∎		
Obesity	∎									∎	
Shortening/bowing of bones		∎					∎	∎	∎		
Ectodermal dysplasia						∎	∎		∎		

*ATD* asphyxiating thoracic dystrophy (Jeune syndrome), *ALSM* Alström syndrome, *BBS* Bardet–Biedl syndrome, *CED* cranioectodermal dysplasia (Sensenbrenner syndrome), *JBTS*, Joubert syndrome, *MKS* Meckel–Gruber syndrome, *NPHP* nephronophthisis, *OFD1* oro-facio-digital syndrome 1, *PKD*42polycystic kidney disease, *SLSN* Senior–Løken syndrome, *SRP* short rib polydactyly syndrome

### Renal cystic dysplasia and other renal phenotypes

Other renal phenotypes have also been associated with ciliary dysfunction. In severe syndromes that affect early human development, such as the Meckel–Gruber syndrome, which is characterized by neural tube defects and many other features, and the short-rib-polydactyly syndrome, fetuses present with cystic renal dysplasia, a congenital renal dysplasia in which the renal cortex is generally cystic, with distension of the terminal ends of the collecting ducts, and the medullary pyramids are poorly developed and demonstrate dysplastic structures and fibrous tissue (Fig. 2b) [3, 27–29]. Other renal phenotypes that have been described in ciliopathies are horseshoe kidneys [29, 30], lobulated kidneys [31], urinary tract infections and anomalies [32], and kidney stones [33]; however, the latter abnormalities are all much less commonly reported and it remains to be shown whether these features are (in part) the result of cilium dysfunction or not.

## Genetics of renal ciliopathies

### Current genetic insights

To date, mutations in roughly 50 genes have been associated with renal ciliopathies (Table 1). Although not all patients with mutations in these genes suffer from renal disease, we have to be aware of the fact that various genes were identified only recently in a few young patients in whom renal disease may still develop, and that a subset of genes have a low-mutation frequency. Improved insights into genotype–phenotype relations are thus warranted for better diagnosis and prognosis, and screening for early signs of renal disease is important in most individuals.

### From SNP microarray analysis to next-generation sequencing

**Fig. 3:**
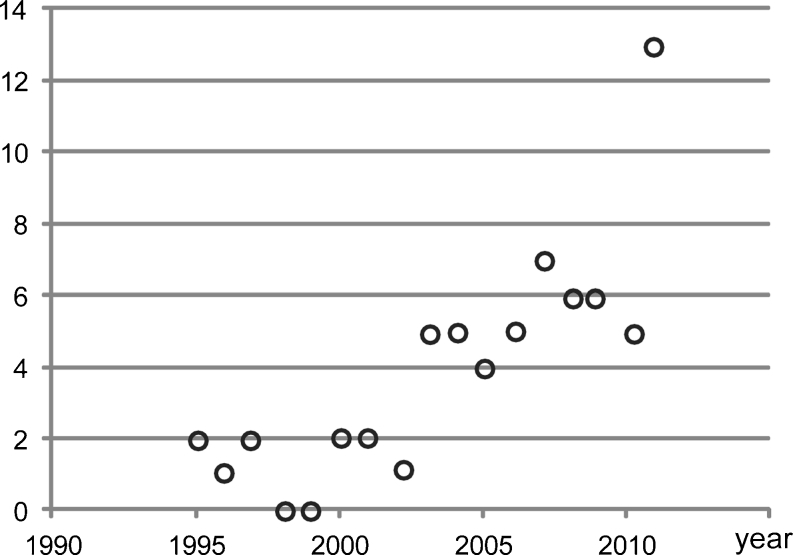
Gene identification for renal ciliopathies in the period from 1995 to 2011. *Open circles* indicate the number of genetic causes that were identified in the corresponding year. X-axis: time in years; y-axis: number of genetic causes

Although gene defects are still identified through linkage analysis with single nucleotide polymorphism (SNP) microarrays followed by candidate sequencing, e.g., *KIF7* associated with acrocallosal, hydrolethalus, and Joubert syndrome [34, 35], *CEP41* associated with Joubert syndrome [36], and *NEK1* associated with short rib polydactyly [29], next-generation sequencing (NGS) techniques are dramatically speeding up gene identification in the ciliopathy field and in the genetics field in general (Fig. 3) [37]. These NGS technologies allow cost-effective and time-efficient gene identification by sequencing large parts or even the full complement of (protein-coding) DNA of a single individual at once. Gene identification can now take a matter of weeks rather than years [38]. Targeted parallel-sequencing of linkage intervals or small groups of genes led to the identification of mutations in the renal ciliopathy genes *B9D1* associated with Meckel–Gruber syndrome [39] and *TMEM237* associated with Joubert syndrome [40], while larger-scale ciliopathy candidate exome (ciliome) sequencing led to the detection of mutations in *SDCCAG8* [41] and *IFT140* [42]. Whole-exome sequencing, whereby all protein-coding DNA (all exons of the genome, 1% of the genome) is sequenced, has also been applied and resulted in the detection of mutations in *WDR35* [22]*, WDR19* [43], and *C5ORF42* [44] as causes of Sensenbrenner and Joubert syndromes respectively. In addition, insights into genotype–phenotype correlations are quickly evolving through parallel-sequencing of a series of known renal ciliopathy genes in large patient cohorts (consisting of roughly 100–500 individuals) [45–47]. This is of great value as this knowledge improves diagnosis, prognosis, and genetic counseling for patients and their relatives.

### Dissection of protein complexes facilitates gene identification

Other methods that contribute to gene identification, albeit indirectly, are state-of-the-art proteomic tools, such as tandem affinity purification and yeast two-hybrid assays, that allow dissection of protein networks [14]. Interaction studies taught us that ciliopathy-associated proteins interconnect in a relational ciliopathy protein complex, a dynamic molecular machine that allows ciliary growth and function [13, 15]. In addition, there is ample evidence to conclude that proteins in the ciliopathy network form sub-clusters, which are often associated with distinctive clinical characteristics when mutated. This is illustrated by the following examples. Many of the “nephrocystins” encoded by the nephronophthisis genes physically link together in a “nephrocystin” protein complex (Fig. 1) [14, 48–51]. Similarly, Jackson showed that this is also the case for proteins that are mutated in Bardet–Biedl syndrome by demonstrating that BBS4 associates with a variety of other BBS proteins (i.e., BBS1, 2, 5, and 7–9), a module that is referred to as the BBSome (Fig. 1) [52]. BBS6, 10, and 12 were later shown to form a smaller protein complex necessary for the assembly of the BBSome [53]. Consistently, skeletal ciliopathies such as Jeune syndrome, Sensenbrenner syndrome, and short rib polydactyly have almost explicitly been correlated with defects in proteins that form IFT protein complexes (Fig. 1). In 2007, mutations in *IFT80* were found to be associated with Jeune syndrome [54], a gene encoding a protein that is part of the multi-subunit IFT-B complex; however, almost all other mutations associated with human skeletal ciliopathies occur in genes encoding proteins that are part of the IFT-A complex involved in retrograde transport, i.e., *IFT122* [23], *WDR35*/*IFT121* [22, 28], *TTC21B*/*IFT139* [55], *WDR19*/*IFT144* [43], *IFT140* [42], *IFT43* [56], and *DYNC2H1* [57], a subunit of the cytoplasmic dynein motor 2. Taken together, these different (interconnected) protein modules of nephrocystins, BBS proteins, and IFTs are associated with somewhat specific, but overlapping phenotypes [13, 15]. The fact that ciliary proteins form an interaction network insinuates that systematic interaction assays for nephronophthisis-associated proteins may reveal novel candidate disease genes. Excitingly, high-throughput tandem affinity purifications for “nephrocystins” and “Meckel–Gruber-associated proteins” have recently been executed in ciliated cells, and have indeed proven to facilitate gene identification when combined with clinical SNP microarray data from families with ciliopathies; mutations in *ATXN10* and *TCTN2* were recently identified as the cause of nephronophthisis and Joubert syndrome through this combination of methods [14].

### Regulatory mechanisms

**Fig. 4:**
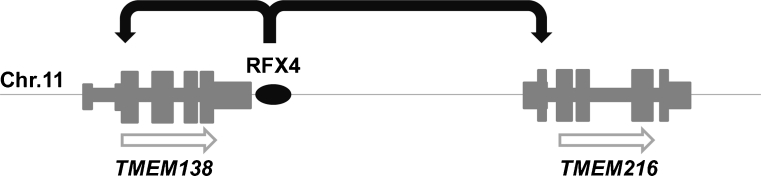
Gene regulation of two adjacent nonhomologous disease genes. The neighboring *TMEM138* and *TMEM216*, mutated in Joubert syndrome, are regulated by transcription factor RFX4, which binds to a noncoding conserved regulatory intergenic region (*black oval*). *Open arrows* indicate that both genes are located on the sense strand of chromosome 11

Although it is still challenging to understand how genes are regulated and which noncoding intergenic regions regulate (ciliary) gene expression, this field is evolving as we speak. The Gleeson group recently published an impressive article in *Science* on gene regulation in Joubert syndrome [58]. As mutations in *TMEM216* explained only half of their families with linkage in the JBTS2 locus, they hypothesized that there must be another disease gene in this region. Excitingly, re-sequencing identified mutations in *TMEM138*, which neighbors *TMEM216* head-to-tail (Fig. 4). Both genes appear to be co-regulated by the ciliary transcription factor regulatory factor X 4 (RFX4), a member of the RFX family that consists of at least seven proteins in humans [59], which binds at a noncoding-conserved intergenic region that lies between *TMEM138* and *TMEM216* (Fig. 4) [58]. It is currently unknown how often nonhomologous, adjacent genes associated with indistinguishable phenotypes share regulatory motifs. As suggested by Gleeson, it will be interesting to determine how transcriptional regulation occurs of the neighboring genes *EVC* and *EVC2*, which are mutated in the ciliopathy Ellis–van Creveld syndrome [60]. Generally, co-expression studies will lead toward new insights into the molecular basis of the genetic disorders and may facilitate gene identification and development of targeted therapies for renal ciliopathies.

## Genotype–phenotype correlations

### Mutation type

Mutations in single ciliary genes are often associated with multiple phenotypes. It is generally believed that the nature of the mutations strongly influences the severity of the resulting phenotype, i.e., truncating mutations are associated with more severe phenotypes than missense mutations. However, although this trend is often observed, there are no clear-cut genotype–phenotype correlations, and the fact that clinical differences are often observed between members of single families indicates that the phenotypes also result from modifier effects [3, 4]. It is particularly striking that roughly 40% of the Joubert syndrome-associated genes, i.e., *CC2D2A*, *CEP290*, *NPHP3*, *RPGRIP1L*, *TCTN2*, *TMEM216*, and *TMEM67*, are also mutated in the closely related, but more severe, Meckel–Gruber syndrome [4]. Similarly, Sensenbrenner and Jeune syndromes appear to be milder presentations of the embryonically lethal short rib polydactyly syndrome as *IFT80, WDR35* and *DYNC2H1* are mutated in both milder and more severe phenotypes [22, 28, 54, 57, 61]. Yet, “mild” phenotypes seem to go beyond Sensenbrenner and Jeune syndromes, given the fact that mutations in the IFT genes *TTC21B* and *WDR19* are also associated with isolated nephronophthisis [43, 55]. Future research will show how broad the associated phenotypic spectrum is for the IFT proteins. With respect to the remaining renal ciliopathy genes allelism, which refers to differing phenotypes from different defects in the same gene, is broader than outlined above, and occurs for 30% of the genes listed in Table 1, which further emphasizes the clinical heterogeneity associated with this group of genes.

### Mutational load

Apart from mutation type, “genetic load,” “modifier effects,” and “oligo-genetic inheritance,” which all refer to the possibility that mutations in more than one gene affect the phenotype, have also been proposed as an explanation for the clinical variability within ciliopathies and within families suffering from these disorders. Mutations in multiple genes have been reported in most renal ciliopathies, initially in Bardet–Biedl syndrome [62, 63] and later throughout the ciliopathy spectrum [55, 64, 65]. In this respect, there is also exciting news on the dosage theory from the PKD field. Although mutations in *PKD1* and *PKD2* are associated with dominant disease, it was recently reported that two hypomorphic *PKD1* alleles may result in ARPKD-like disease in utero [66]. As the parents of these fetuses had a negative PKD family history, this tells us that it is important to be aware of the possibility that multiple mutations in ADPKD-associated genes can explain renal cystic disease in families with apparently ARPKD. Several other papers describing severe manifestations of PKD also demonstrated that multiple mutations may be present in PKD genes and other genes such as *TSC2*/*HNF1β* [67–69]. The “mutational load” theory is thus not only applicable to recessive renal ciliopathies, but also to dominant renal cystic disorders.

## New ciliopathies

New ciliopathies can be uncovered in different ways. Baker and Beales accurately predicted that various syndromes such as Jeune asphyxiating thoracic dystrophy, Sensenbrenner syndrome (also known as cranioectodermal dysplasia), and Saldino–Mainzer syndrome belong to the ciliopathy spectrum [20]. Their analysis was based on screening for (multiple) classic ciliopathy features in the Online Mendelian Inheritance in Man (OMIM) clinical database. Besides comparing human phenotypes, we can also extract predictive markers for human disease from studies with mouse mutants; there are for instance clues from a conditional murine *Kif3a* mutant that frontonasal dysplasia could be the result of ciliary dysfunction [70–72]. Finally, identification of novel ciliary functions for proteins associated with human disease may reveal that the molecular cause of disease may be (in part) due to ciliary disruption, thereby opening avenues for development of targeted therapies. A recent example is a publication from Coon et al. on Lowe syndrome [73]. This syndrome is a cerebrorenal developmental disorder that is characterized by Dent’s disease in the kidney, a renal proximal tubulopathy. The association of defective cilia with this syndrome and the fact that Dent’s disease has not previously been associated with ciliary dysfunction raises the question whether there are more patients with this renal phenotype with mutations in different genes that encode proteins involved in the biology of renal cilia.

## Next-generation sequencing and clinical perspectives

Currently, NGS is mostly used for research purposes to identify novel disease genes and to gain more insights into genotype–phenotype correlations in a time- and cost-effective effort. The power of NGS has proven itself in research laboratories, and in the coming years this technology will be implemented in DNA diagnostic laboratories throughout the world. Whereas disease genes are currently stepwise Sanger sequenced in diagnostics based on an educated guess at the best candidate gene, whereby clinical phenotype, mutation frequency, and ethnic origin are considered, unbiased mutation screening through NGS is expected to be much more effective [3]. Although NGS will improve diagnosis, prognosis, and genetic counseling for patients in daily clinical practice, there are also challenges for the implementation of this technology in DNA diagnostics [74]. Data interpretation must be focused on finding mutations in known genes, thus requiring the development of new software for data analysis. Excellent bioinformaticians and infrastructures are a necessity for NGS data management and analysis. The latter is a bottleneck in research, and will be an even more prominent problem in DNA diagnostics, as the accuracy of mutation detection is more important in the clinic, raising questions on how to handle poor sequence coverage for selected genomic regions in a diagnostic setting. Aside from the technical challenges, the ethical implications of NGS are enormous [75, 76]. For instance, NGS may identify mutations in genes unrelated to the studied disease, which has major implications for patients and their relatives. Concerning renal ciliopathies, should we sequence exomes or a selected set of known ciliopathy genes to avoid the latter? What to do with variants of unknown significance? What should be the content of a consent form, especially with respect to unanticipated mutations? Whose consent should be asked, given that NGS findings could also have a major impact on the lives of family members? These are just a few of the long list of ethical issues that remain to be resolved.

## Roads to therapy

Once end-stage renal disease develops, patients with renal ciliopathies currently depend on invasive therapies such as hemodialysis or renal replacement strategies, which have a major impact on the quality of life for patients and their relatives. The development of targeted therapies is thus warranted. In this respect significant progress has been made in the (AD)PKD field. Several clinical trials are underway, some are finished, and potential drug targets are continuously being identified in rodent models [77–84]. Because the PKD- and nephronophthisis-associated genes are involved in similar ciliary pathways, PKD treatment may eventually also appear to be valuable for nephronophthisis patients. Targeted therapy development for nephronophthisis has fallen behind in comparison to that for PKD, likely because of the lower prevalence and enormous genetic heterogeneity associated with this disorder. Fortunately, the NGS-accelerated identification of genetic defects in nephronophthisis opens a window of opportunity for the development of (personalized) therapies, as insights into disease mechanisms are increased and targeted drug screens become possible. Notably, cost- and time-effective small molecule screening in zebrafish is expected to contribute to drug discovery [85, 86] and various mouse models are already being tested for selected compounds [87]. Furthermore, the development of the induced pluripotent stem cell (iPSC) technology allows drug screening in a patient’s own cells and may hold promise for future tissue regeneration therapy [88, 89]. Although many studies are focused on the treatment and prevention of PKD, the first stones in the path toward targeted therapy development for nephronophthisis are only just beginning to be laid. Yet, with the recent development of NGS we now have the chance to remedy this situation by rapidly exposing potential treatment targets, and by using this information for personalized medicine.

## Conclusions

We are well on our way to identifying the genetic mechanisms underlying the renal ciliopathies. NGS methods are accelerating this process enormously, and massive parallel-sequencing genetic tests will soon be available for routine diagnostic screening. The availability of such tests will improve diagnostics, prognosis, and genetic counseling tremendously; however, challenges in bioinformatic analysis and variant interpretation remain, as well as the requirement of strict ethical regulations. Besides genetic progress, molecular studies in ciliated (patient) cell lines and model organisms expanded our insights of the disease mechanisms of nephronophthisis and associated disorders. The next challenge is to use this genetic and molecular knowledge toward the development of targeted (personalized) therapies to delay and preferably prevent the progressive degenerative effects of nephronophthisis in patients.
